# Tissue cytokines in chickens from lines selected for high or low humoral antibody responses, given supplemental *Limosilactobacillus reuteri* and challenged with *Histomonas meleagridis*

**DOI:** 10.3389/fphys.2023.1294560

**Published:** 2024-01-04

**Authors:** Frank W. Edens, Paul B. Siegel, Robert B. Beckstead, Christa F. Honaker, Dellila Hodgson

**Affiliations:** ^1^ Prestage Department of Poultry Science, North Carolina State University, Raleigh, NC, United States; ^2^ School of Animal Sciences, Virginia Polytechnic Institute and State University, Blacksburg, VA, United States

**Keywords:** *Limosilactobacillus reuteri*, *Histomonas meleagridis*, chickens, cytokines, antibody selected lines

## Abstract

*Histomonas meleagridis*, a protozoan parasite, induces blackhead disease (histomoniasis) in poultry. During hatching, chicks from lines divergently selected for high (HAS) and low (LAS) antibody responses to sheep red blood cells were divided into two groups, each of HAS and LAS, and placed in pens with wood shavings as litter. Feed and water were allowed *ad libitum*. Half of the chicks from each line had *Limosilactobacillus reuteri (L. reuteri)* inoculated to their drinking water. On day 18, all chicks were given a transcloacal inoculation of 100,000 *H. meleagridis* cells. Then, 10 days later, they were euthanized, followed by collection of tissues from the brain, cecal tonsil, ceca, liver, thymus, and spleen for qPCR analyses of cytokines involved in immunological development. Changes in cytokine expressions were most numerous in the cecal tonsil, ceca, and liver. In the absence of a functional medication for control of histomoniasis, *L. reuteri* and/or its secretory product, reuterin, might serve, in some genetic populations, as a means to reduce the impact of histomoniasis in chickens. The data demonstrate that *L. reuteri* treatment had tissue specificity between the two genetic lines, in which the effects were targeted primarily toward the cecal tonsil, ceca, and liver, which are the primary tissue targets of the parasite (*H. meleagridis*), as well as the thymus and spleen. However, interactions among main effects reflect that responses to inflammatory markers observed in tissues for one genetic line may not be observed in another.

## Introduction

Since the 1890s, the poultry industry has been concerned with the trichomonad *Histomonas meleagridis* (*H. meleagridis*), which, in gallinaceous birds, induces a severe parasitic disease called histomoniasis, commonly known as blackhead disease (Cushman, 1893). The histomonads generally affect the ceca and liver, causing extreme mortality in turkeys and less serious infections in chickens (Beckstead, 2019). Blackhead disease targets the liver, inducing hepatic failure called enterohepatitis (Smith, 1895; Smith, 1915). Infectious enterohepatitis is characterized by necrotic foci in the liver and extreme inflammation and ulceration in the ceca, which are associated with increased mortality, especially in turkeys, with very few individuals surviving the infection (Smith, 1895; Smith, 1915).

Histomoniasis in chickens ranges in severity from mild to severe. Although mortality can be as high as 70% with morbidity up to 90%, some strains of infected chickens can often be asymptomatic (Huber et al., 2006). In turkeys, flock morbidity and mortality can reach 100%. Histomoniasis is poorly understood in both chickens and turkeys (Beckstead, 2019; Liebhart and Hess, 2020), and well-defined reasons for the differences between them in susceptibility and responses are not apparent. Cytokine production and the phenotype of *H. meleagridis*-specific T cells in infected chickens revealed a systemic protective type 1 T-cell-related phagocytic immune response against the parasite, which was focused on INF-γ-producing CD4^+^ and CD4^−^–CD8 β-splenocytes (Spellberg and Edwards, 2001). However, there was no indication of a systemic type 2 antibody-mediated immune response involving IL-10 and IL-13 cytokine induction, whereas in rodents, they are involved in type 2 cytokine responses associated with resistance to most gastrointestinal nematodes (Wynn, 2001). Research comparing, simultaneously, immunologically active cytokine production in both chickens and turkeys revealed that, in contrast to chickens, turkeys tended to have a much slower immune response to the *H. meleagridis* challenge (Mitra et al., 2018).

Powell et al. (2009) observed that the chicken mounted a more effective cecal innate response via elevated expressions of IL-1β, CXCLi2, and IL-6 mRNA, which were associated with better control of parasite numbers. However, the turkey failed to mount such an effective innate response in the cecal tonsils, which was correlated with greater numbers of histomonads migrating to the liver. When the histomonads began to colonize the liver, there was an apparent sustained, uncontrolled immune response involving infiltration of CD4^+^, CD8α+, CD28^+^, and CD44^+^ cells (Mitra et al., 2017). In chickens, however, cellular influx was better controlled in the liver where there was an upregulation of mRNA for IL-1β, CXCLi2, IFN-c, IL-13, IL-4, and IL-10. Expression levels of mRNA of the chicken and turkey β-defensin (AvBD2) suggested that this response was not limited to the cytokines. The massive influx of the CD4^+^, CD8α+, CD28^+^, and CD44^+^ cells into the liver was more pronounced in the turkey and was related to a decrease in those cell phenotypes from turkey than chicken spleens. Furthermore, antibody levels were greater in the *H. meleagridis*-challenged chickens than those in turkeys (Powell et al., 2009), which supports the hypothesis of a greater adaptive immune capability in chickens than turkeys.

Lines of chickens originating from a common founder population and selected for either high (HAS) or low (LAS) antibody response to a sheep red blood cell challenge (Gross et al., 1980; Gross and Siegel, 1980; Siegel and Gross, 1980; Nolin et al., 2023) have differential responses to various infectious disease challenges (Gross et al., 2002). HAS shows the potential to produce greater antibody titers, mobilize a heterophil response as a first-line defense, and is more resistant than LAS when subjected to either the *Mycoplasma* or *Escherichia coli* challenge (Gross et al., 1988; 2002). On the other hand, LAS was more resistant than HAS to the development of splenomegaly, following an avian adenovirus group II challenge (Gross et al., 1988).

Observations on the immunological potential of HAS and LAS showed that LAS was more resistant to invasion by extracellular pathogens, whereas HAS was more resistant to infection by intracellular pathogens (Sumners et al., 2012). In addition, the major histocompatibility complex (MHC) was fixed at B13B13 in LAS and B21B21 in HAS by the 13th generation of divergent selection (Dunnington et al., 1996). MHC-B haplotype chickens are a valuable resource for studying the genetic effects of pathogen resistance and susceptibility (Gehad et al., 1999). Martin et al. (1986) reported that HAS was more resistant to *Eimeria tenella* infection than LAS, with the degree of infection associated with the blood group background.

The response of HAS and LAS lines to *H. meleagridis* infection has not been assessed. Although the pathogenesis mechanisms of *H. meleagridis* remain unidentified, we know that it does not invade host cells, thus making it an extracellular pathogen. This suggests that LAS should be more resistant to *H. meleagridis* than HAS. In addition, much of the damage exerted by this protozoan parasite can be attributed to cysteine proteases (CPs), which cause tissue damage associated with cytolysis, cytoadherence, hemolysis, and cytotoxicity (Mazumdar et al., 2017; Wei et al., 2020). Although, to the best of our knowledge, identification of virulence gene(s) has not been reported for *H. meleagridis’* involvement of the parasite’s pathogenic mechanism; the expression of virulence genesis believed to play a crucial role (Mazumdar et al., 2017; Wei et al., 2020).

Reported here are results of a *H. meleagridis* challenge to HAS and LAS chickens at 18 days post-hatch. *Limosilactobacillus reuteri* (formerly called *Lactobacillus reuteri*; *L. reuteri*) was added to the drinking water of chicks in half of the pens. Our objective was to compare differential expressions of immunologically associated cytokines among different tissues, which included the brain, cecal tonsil, ceca, liver, thymus, and spleen in treated and untreated chickens from these two genetic lines, which originated from a common founder population.

## Materials and methods

### Animal welfare

All procedures in the experiment were approved by the Institutional Animal Care and Use Committee at North Carolina State University, Raleigh, NC 27695.

### Animal husbandry

Eggs from the S45 generation of HAS and LAS chicken lines (Gross and Siegel, 1980; Siegel and Gross, 1980) were hatched at the North Carolina Agricultural Research Service Chicken Educational Field Laboratory at North Carolina State University. Breeders were of the same age and raised as contemporaries. During hatching, both male and female chicks were wing-banded and placed into four pens, with two per genetic line, housing 30 chicks per pen. The pens consisted of 7.5 cm of fresh pine wood shavings over concrete floors. The initial floor brooding temperature was set at 35°C and reduced gradually to 27°C by day 15 and maintained at that temperature until day 21. From day 21 until termination at day 28, the ambient temperature was maintained at 24°C. During week 1, the chicks were given 23 h of light/day; from day 7 through day 13, light was provided at 16 h/day, and from day 14 through day 28, light was provided at 12 h/day. Light intensity was 0.5 foot-candles at the floor level. The diet fed was in a mashed form and was formulated to provide 19% CP and dietary energy at 12.373 MJ/kg, which met the National Research Council (1994) dietary requirements. Feed, in hanging hoppers, was available *ad libitum*. Water was available *ad libitum* in plastic drinkers.

### Procedures

On day 18, each chick was given a transcloacal inoculation of 100,000 cells of the wild-type Buford strain of *H. meleagridis* (Wei et al., 2020; Beer et al., 2022), originally isolated from infected chickens in Georgia and maintained in our laboratory. *H. meleagridis* was inoculated in a volume of 1 mL in Dwyer’s growth medium (Hauck et al., 2010). Each chick was suspended upside down for a period of 2 min to ensure that a sufficient number of histomonads were passively swept up the large intestine by reverse peristalsis to enter the ceca. *L. reuteri* (BioGaia Biologics AB, Stockholm, Sweden), a water-delivered probiotic, was added to the water troughs of the appropriate pens (1–HAS + *H. meleagridis*, 2–HAS + *H. meleagridis* + *L. reuteri*, 3–LAS + *H. meleagridis*, and 4–LAS + *H. meleagridis* + *L. reuteri)* every 48 h as a lyophilized product dispersed in skim milk powder to provide at least 1,052,631 cfu/d/chick in non-chlorinated well water. The drinkers were washed daily.

On day 28, individual chicks were euthanized by cervical dislocation, followed by the opening of the body cavity, examined for lesions associated with histomoniasis in the ceca and liver, followed by rapid collection of rice grain-sized samples from the cecal tonsil, mid-ceca, spleen, liver, thymus, and a sagittal half of the brain. All tissues were immersed in 20 mL of Ambion RNALater (Thermo Fisher Scientific, Raleigh, NC), maintained at room temperature for 24 h, and then placed in a −20°C freezer until processed for RNA isolation.

The amount of mRNA present in a tissue is related to overall gene expression, and to standardize the amount of RNA in a sample, we used glyceraldehyde-3-phosphate dehydrogenase (GAPDH) as the internal standard (house-keeping) gene. Total RNA was isolated from tissues, following the manufacturer’s instructions, using Qiagen’s RNeasy Lipid Tissue Mini kit (QIAGEN, Valencia, CA) for brain and liver tissues, and Qiagen’s RNeasy Mini kit, following the manufacturer’s protocol for non-fatty tissues. The RNA quality was assessed using a NanoDrop 2000 spectrophotometer (Thermo Fisher Scientific, Raleigh, NC). Total RNA was quantified using BioTek’s Synergy HTX multimode reader (model #14651, Cambridge Scientific, Watertown, MA). qRT-PCR was conducted using the Eppendorf Vapo Protect (Eppendorf AG, Hamburg, Germany). Primers for IL-2, IL-8, IL-10, IL-13, IL-15, IL-16, IL-18, iNOS, and LITAF were obtained from Integrated DNA Technologies (Coralville, IA). An ethogram showing primary functions of the interleukins and cytok"ines assayed is presented in Table 1. Selection of primers shown in Table 2 was based on previous research (Sumners et al., 2012) to ensure compatibility. qPCR was performed using a 3-step PCR protocol; 10-min initial denaturation at 95°C, followed by 40 cycles at 95°C (denaturation), 60°C (annealing), and 72°C (elongation), each step in the cycle lasting 30 s except for the last elongation cycle that was for a period of 10 min. An amount of 1,000 ng of cDNA was used in a 20-µL reaction. The primers were added at a concentration of 2.5 nM for the forward reaction and 2.5 nM for the reverse reaction.

**TABLE 1:** Ethogram for cytokines and interleukins measured herein.

Cytokine/Interleukin	Description
IL-2	Interleukin-2, upregulates regulatory T-cell production
IL-8	Interleukin-8, upregulates inflammation
IL-10	Interleukin-10, macrophage stimulation
IL-13	Interleukin-13, inhibition of pro-inflammatory cytokines
IL-15	Interleukin-15, similar to IL-2; promotes immunoglobulin production
IL-16	Interleukin-16, lymphocyte chemo-attractant for immuno-active cells
IL-18	Interleukin-18, pro-inflammatory; IFN-ɣ-inducing factor
iNOS	Inducible nitric oxide synthase, increases NO from macrophages; cytotoxic
LITAF	Lipopolysaccharide-induced tumor necrosis-α-factor, regulates TNF-α gene expression during gut parasitic infections; pro-inflammation

**TABLE 2:** Chicken primer sequences (from Sumners et al., 2012).

Gene	Primer^a^	Accession number
Interleukin (IL)-2	For: 5′– CGA​GCT​CTA​CAC​ACC​AAC​TGA​GA—3′	NM_204153
	Rev: 5′– CCA​GGT​AAC​ACT​GCA​GAG​TTT​GC—3′	
IL-8	For: 5′– TCC​TGG​TTT​CAG​CTG​CTC​TGT—3′	NM_205498
	Rev: 5′– CGC​AGC​TCA​TTC​CCC​ATC​T—3′	
IL-10	For: 5′– CGC​TGT​CAC​CGC​TTC​TTC​A—3′	NM_001004414
	Rev: 5′– CGT​CTC​CTT​GAT​CTG​CTT​GAT​G—3′	
IL-13	For: 5′– CAT​GAC​CGA​CTG​CAA​GAA​GGA—3′	NM_001007085
	Rev: 5′– CCG​TGC​AGG​CTC​TTC​AGA​CT—3′	
IL-15	For: 5′– GAC​TAA​CCA​TCT​TCT​TCC​TAT​GTG​CTT—3′	AF139097
	Rev: 5′– AGA​ACG​TCT​GAC​CAC​TTA​CAG​TGA​TT—3′	
Il-16	For: 5′– GGA​ACA​AAG​CAG​CCC​AGT​TC—3′	NM_204352
	Rev: 5′– GGC​TGT​GGT​GTG​CAC​CTG​TA—3′	
IL-18	For: 5′– AGG​TGA​AAT​CTG​GCA​GTG​GAA​T—3′	NM_204608
	Rev: 5′– TGAAGGCGCGGTGGTTT—3′	
iNOS^b^	For: 5′– CCT​GTA​CTG​AAG​GTG​GCT​ATT​GG—3′	D85422
	Rev: 5′– AGG​CCT​GTG​AGA​GTG​TGC​AA—3′	
LITAF^c^	For: 5′– TGT​TCT​ATG​ACC​GCC​CAG​TTC—3′	AY765397
	Rev: 5′– AGA​CGT​GTC​ACG​ATC​ATC​TGG​TTA—3′	

^a^ For, forward primer; Rev, reverse primer; sequences were generated using Primer Express 3.0.

^b^ Inducible nitric oxide synthase.

^c^ Lipopolysaccharide-induced tumor necrosis factor- α.

### Data analyses

Analyses of variance associated with the general linear models of the statistical analysis system (SAS Institute, Inc, 2013) were used to test for effects due to genetic lines (HAS and LAS), treatments (*L. reuteri* and no *L. reuteri*), and tissues (the brain, cecal tonsil, ceca, liver, thymus, and spleen), as well as interactions among them for IL-2, IL-8, IL-10, IL-13, IL-15, IL-16, IL-18, iNOS, and LITAF. Lines, treatments, and tissues were considered as fixed effects. The statistical model was Y*ijkl* = *μ* + Li + Nj + Tk + (LN)ij + (LT)ik + (NT)jk + (LNT)ijk + eijkl, where i represents 1, 2 lines; j represents 1, 2 treatments; k represents 1, 2 tissues; and I represents 1, 2….*n* individuals. When interactions among main variables were significant, the data were analyzed within pairs of variables. When tissue means were significantly different, they were separated by Duncan’s multiple range test. Statistical significance was accepted at *p* ≤ 0.05.

## Results

### Biological responses of HAS and LAS chicks

The results are presented by combinations of main effects and interactions to enhance descriptions of the diversity of lines, tissues, and inflammatory markers. A lower value for a marker reflects a greater inflammatory response.

### Base line values

Prior to treatment and challenge on day 18, a sample of each tissue from the lines was assayed for each of the cytokines. The HAS means for the liver, the main target organ for histomonads, were 0.58, 0.67, 0.57, 0.58, 0.79, 0.68, 0.75, 0.70, and 0.71 for IL-2, IL-8, IL-10, IL-13, IL-15, IL-16, IL-18, iNOS, and LITAF, respectively. For LAS, the respective means were 0.55, 0.67, 0.56, 0.61, 0.76, 0.70, 0.71, 0.68, and 0.74.

### Histomonas meleagridis

There was only one chick, a male HAS, that died 9 days post-challenge. It had a cecal core; however, there were no visible hepatic lesions. At 10 days post-challenge, when all chicks were euthanized for tissue collection and determination of lesion scores for histomoniasis, no chicks showed any signs of infection (cecal inflammation and/or hepatic lesions).

### *Limosilactobacillus reuteri* effect within the lines

Comparisons of qPCR means for each marker, segregated by a line and whether or not *L. reuteri* was present in their drinking water (Table 3), showed that IL-13 expression was significantly elevated in HAS but not LAS chicks, when *L. reuteri* was inoculated. Expression levels of IL-16 and IL-18 were elevated in LAS, when *L. reuteri* was inoculated, but not in HAS. There were no differences between treated and control chicks within either line for the other inflammatory markers measured.

**TABLE 3:** Means^a^ and standard errors of qPCR results for inflammatory markers for treatment within each line.

Marker	HAS^b^			LAS^b^		
	L^c^		N^c^	L^c^		N^c^
IL-2	0.56 ± 0.01	*	0.57 ± 0.01	0.55 ± 0.01	* *	0.55 ± 0.01
IL-8	0.67 ± 0.01		0.67 ± 0.01	0.66 ± 0.01		0.68 ± 0.02
IL-10	0.59 ± 0.01		0.61 ± 0.01	0.60 ± 0.01		0.59 ± 0.01
IL-13	0.55 ± 0.02		0.59 ± 0.01	0.55 ± 0.01		0.57 ± 0.01
IL-15	0.71 ± 0.02		0.74 ± 0.01	0.68 ± 0.02		0.71 ± 0.01
IL-16	0.73 ± 0.02		0.76 ± 0.01	0.72 ± 0.01		0.79 ± 0.02
IL-18	0.74 ± 0.01		0.73 ± 0.01	0.71 ± 0.01		0.74 ± 0.01
iNOS	0.77 ± 0.01		0.76 ± 0.01	0.77 ± 0.01		0.77 ± 0.01
LITAF	0.78 ± 0.01		0.79 ± 0.01	0.76 ± 0.01		0.78 ± 0.01

* Means between treatments, within a line, are different (*p* ≤ 0.05).

^a^ (raw number/GAPDH)^-1^.

^b^ HAS, high-antibody line; LAS, low-antibody line.

^c^ L, *L. reuteri* added; N, no *Lactobacillus* (n = 36, except LAS L IL-2, n = 41; LAS N IL-2, n = 30; LAS L IL-8, n = 42)

### Line effects on tissue cytokines

Comparisons between lines among the six tissues for the nine inflammatory markers (Table 4), where there was no line-by-tissue interaction, showed several significant line differences depending on the tissue assay. For IL-2, there was significant upregulation in the LAS ceca and HAS liver. IL-15 was upregulated in the LAS ceca and spleen. IL-18 was upregulated in the LAS brain and ceca. The iNOS expression rate was upregulated only in the HAS cecal tonsil, while LITAF was upregulated in the LAS brain and ceca.

**TABLE 4:** Means^a^ and standard errors of qPCR results for inflammatory markers, comparing lines within each tissue.

Marker	Line^b^	Tissue											
		Brain		Cecal tonsil		Ceca		Liver		Thymus		Spleen	
IL-2	HAS LAS	0.53 ± 0.010.53 ± 0.01	* *	0.57 ± 0.01 0.56 ± 0.01	* *	0.61 ± 0.01 0.57 ± 0.01	* * * *	0.53 ± 0.01 0.58 ± 0.01	* * *	**0.54 ± 0.01** **0.48 ± 0.01**	* * *	0.59 ± 0.01 0.57 ± 0.01	* * *
IL-8	HAS LAS	0.66 ± 0.02 0.68 ± 0.01		**0.71 ± 0.02** **0.72 ± 0.02**		0.73 ± 0.02 0.71 ± 0.03		**0.64 ± 0.01** **0.67 ± 0.01**		**0.57 ± 0.01** **0.52 ± 0.01**		0.70 ± 0.02 0.71 ± 0.01	
IL-10	HAS LAS	0.65 ± 0.02 0.63 ± 0.01		0.63 ± 0.02 0.65 ± 0.02		0.65 ± 0.01 0.62 ± 0.02		**0.54 ± 0.01** **0.56 ± 0.01**		**0.57 ± 0.01** **0.52 ± 0.01**		0.59 ± 0.01 0.59 ± 0.01	
IL-13	HA LAS	0.55 ± 0.02 0.56 ± 0.01		**0.59 ± 0.01** **0.57 ± 0.02**		**0.57 ± 0.02** **0.59 ± 0.01**		**0.53 ± 0.01** **0.56 ± 0.01**		0.57 ± 0.03 0.51 ± 0.02		**0.62 ± 0.02** **0.58 ± 0.01**	
IL-15	HAS LAS	0.67 ± 0.02 0.68 ± 0.02		**0.77 ± 0.01** **0.71 ± 0.02**		0.76 ± 0.01 0.71 ± 0.02		**0.73 ± 0.02** **0.77 ± 0.01**		0.62 ± 0.03 0.56 ± 0.02		0.80 ± 0.02 0.75 ± 0.02	
IL-16	HAS LAS	0.78 ± 0.04 0.82 ± 0.03		0.78 ± 0.01 0.74 ± 0.02		0.72 ± 0.03 0.71 ± 0.03		0.67 ± 0.01 0.69 ± 0.01		0.74 ± 0.04 0.67 ± 0.02		**0.78 ± 0.03** **0.85 ± 0.02**	
IL-18	HAS LAS	0.75 ± 0.01 0.72 ± 0.01		0.74 ± 0.01 0.72 ± 0.01		0.78 ± 0.01 0.72 ± 0.02		**0.68 ± 0.01** **0.71 ± 0.01**		0.71 ± 0.01 0.69 ± 0.02		0.74 ± 0.01 0.76 ± 0.01	
iNOS	HAS LAS	0.77 ± 0.02 0.72 ± 0.02		0.82 ± 0.01 0.86 ± 0.02		0.84 ± 0.01 0.83 ± 0.02		**0.67 ± 0.01** **0.69 ± 0.01**		0.76 ± 0.01 0.76 ± 0.02		**0.74 ± 0.01** **0.76 ± 0.01**	
LITAF	HAS LAS	0.77 ± 0.01 0.74 ± 0.01		0.84 ± 0.01 0.82 ± 0.02		0.83 ± 0.01 0.78 ± 0.02		**0.72 ± 0.02** **0.75 ± 0.01**		0.75 ± 0.01 0.73 ± 0.02		0.78 ± 0.01 0.80 ± 0.01	

**Bold type** = line-by-tissue interaction (*p* ≤ 0.05).

* Means between lines, within a tissue, are different (*p* ≤ 0.05).

^a^ (raw number/GAPDH)^-1^.

^b^ HAS, high-antibody line; LAS, low-antibody line (n = 12, except LAS; thymus IL-2, n = 11).

### Treatment effects on tissue cytokines

The effects of *L. reuteri* probiotics in drinking water on cytokine expression, where there were no interactions, are presented in Table 5. In the brain, *L. reuteri* in drinking water increased IL-2 expression, but LITAF expression was reduced by the probiotic treatment. In the cecal tonsil, the expression levels of IL-2, IL-10, and IL-16 were upregulated by the *L. reuteri* treatment. However, iNOS expression was decreased by *L. reuteri*. In the ceca, *L. reuteri* induced higher expression of IL-16, while IL-10 exhibited lower expression. In the liver, *L. reuteri* treatment had no effect on IL-2 and IL-16; however, interactions, to be described later, were evident for seven of the nine inflammatory markers. In the spleen, *L. reuteri* was associated with significantly reduced expression of IL-15, while in thymic tissue, *L. reuteri* induced higher expression for both IL-18 and LITAF.

**TABLE 5:** Means^a^ and standard errors of qPCR results for inflammatory markers, comparing treatments within each tissue.

Marker	Treatment^b^	Tissue											
		Brain		Cecal tonsil		Ceca		Liver		Thymus		Spleen	
IL-2	L N	0.52 ± 0.01 0.55 ± 0.01	* * *	0.55 ± 0.01 0.58 ± 0.01	* * * * * * *	0.60 ± 0.01 0.58 ± 0.01	* * *	0.56 ± 0.01 0.56 ± 0.01	* * * * * *	**0.51 ± 0.01** **0.52 ± 0.02**	* * *	0.58 ± 0.01 0.59 ± 0.02	* *
IL-8	L N	0.67 ± 0.01 0.67 ± 0.02		**0.69 ± 0.01** **0.74 ± 0.02**		0.72 ± 0.03 0.72 ± 0.02		**0.64 ± 0.01** **0.67 ± 0.01**		**0.54 ± 0.01** **0.55 ± 0.02**		0.71 ± 0.01 0.70 ± 0.02	
IL-10	L N	0.64 ± 0.01 0.65 ± 0.02		0.61 ± 0.01 0.67 ± 0.02		0.66 ± 0.01 0.60 ± 0.02		**0.53 ± 0.01** **0.57 ± 0.01**		**0.56 ± 0.01** **0.54 ± 0.02**		0.58 ± 0.01 0.60 ± 0.02	
IL-13	L N	0.54 ± 0.02 0.58 ± 0.01		**0.55 ± 0.02** **0.62 ± 0.01**		**0.56 ± 0.01** **0.60 ± 0.01**		**0.53 ± 0.01** **0.56 ± 0.01**		0.51 ± 0.03 0.58 ± 0.02		**0.64 ± 0.02** **0.55 ± 0.01**	
IL-15	L N	0.65 ± 0.02 0.70 ± 0.01		**0.69 ± 0.02** **0.80 ± 0.01**		0.72 ± 0.02 0.75 ± 0.01		**0.73 ± 0.02** **0.77 ± 0.01**		0.56 ± 0.03 0.62 ± 0.01		0.81 ± 0.02 0.74 ± 0.01	
IL-16	L N	0.80 ± 0.04 0.81 ± 0.03		0.73 ± 0.02 0.79 ± 0.01		0.66 ± 0.02 0.77 ± 0.02		0.68 ± 0.01 0.69 ± 0.01		0.68 ± 0.04 0.73 ± 0.02		**0.80 ± 0.01** **0.84 ± 0.04**	
IL-18	L N	0.74 ± 0.01 0.73 ± 0.01		0.73 ± 0.02 0.74 ± 0.01		0.76 ± 0.02 0.75 ± 0.02		**0.69 ± 0.01** **0.71 ± 0.01**		0.68 ± 0.02 0.73 ± 0.01		0.74 ± 0.01 0.77 ± 0.02	
iNOS	L N	0.77 ± 0.03 0.72 ± 0.01		0.87 ± 0.01 0.81 ± 0.01		0.82 ± 0.01 0.84 ± 0.02		**0.67 ± 0.01** **0.70 ± 0.01**		0.75 ± 0.02 0.77 ± 0.01		**0.75 ± 0.01** **0.75 ± 0.02**	
LITAF	L N	0.77 ± 0.01 0.74 ± 0.01		0.81 ± 0.02 0.85 ± 0.02		0.81 ± 0.01 0.80 ± 0.02		**0.72 ± 0.02** **0.76 ± 0.01**		0.71 ± 0.02 0.78 ± 0.01		0.78 ± 0.01 0.81 ± 0.02	

**Bold type** = treatment by tissue interaction (*p* ≤ 0.05).

* Means between treatments, within a tissue, are different (*p* ≤ 0.05).

^a^ (raw number/GAPDH)^-1^.

^b^ L, *L. reuteri* added (n = 13); N, no *Lactobacillus* (n = 11).

### Means for inflammatory markers with a significant line-by-treatment interaction

Overall analyses of line-by-tissue and treatment-by-tissue interactions demonstrate the complexity of selecting tissues to be assayed and the genetic population. Means for significant line-by-treatment interactions are depicted as emboldened values in Tables 4 and 5 and are summarized in Table 6.

**TABLE 6:** Means^a^ of qPCR results for inflammatory markers where there was a significant line-by-treatment interaction.

		HAS^b^		LAS^b^	
Tissue	Marker	L^c^	N^c^	L^c^	N^c^
Cecal tonsil	IL-8	0.71^b^	0.70b^c^	0.67^c^	0.78^a^
	IL-13	0.58^b^	0.61^a^	0.52^c^	0.63^a^
	IL-15	0.74^b^	0.80^a^	0.65^c^	0.79^a^
Ceca	IL-13	0.53^c^	0.61^a^	0.58^b^	0.59^ab^
Liver	IL-8	0.60^b^	0.68^a^	0.67^a^	0.67^a^
	IL-10	0.50^b^	0.58^a^	0.56^a^	0.57^a^
	IL-13	0.50^b^	0.56^a^	0.55^a^	0.57^a^
	IL-15	0.68^b^	0.78^a^	0.78^a^	0.76^a^
	IL-18	0.65^b^	0.71^a^	0.71^a^	0.70^a^
	iNOS	0.63^b^	0.71^a^	0.69^a^	0.69^a^
	LITAF	0.67^c^	0.78^a^	0.76^ab^	0.74^b^
Thymus	IL-2	0.51^b^	0.57^a^	0.51^b^	0.45^c^
	IL-8	0.54^b^	0.60^a^	0.54^b^	0.50^c^
	IL-10	0.56^b^	0.58^a^	0.55^b^	0.48^c^
Spleen	IL-13	0.68^a^	0.55^c^	0.60^b^	0.55^c^
	IL-16	0.81^b^	0.76^c^	0.79^b^	0.94^a^
	iNOS	0.76^b^	0.71^c^	0.74^c^	0.79^a^

Means with different superscripts, within a row, are different (*p* ≤ 0.05).

^a^ (raw number/GAPDH)^−1^.

^b^ HAS, high-antibody line; LAS, low-antibody line.

^c^ L, *L. reuteri* added; N, no *L. reuteri*.

For cecal tonsils, there were significant interactions involving IL-8, IL-13, and IL-15. For IL-8, LAS chickens responded significantly to the probiotic treatment, exhibiting greater expression than LAS chicks with no probiotic and HAS with probiotics in their drinking water. LAS with probiotics had significantly higher expressions of IL-13 and IL-15 than all other treatment groups. Overall, HAS with probiotics had higher IL-13 and IL-15 expression levels than controls for both lines.

There was a significant line-by-treatment interaction for IL-13 for the ceca. HAS with probiotics had a higher expression than other groups. However, in LAS, there was no difference between treatments. In the liver, interactions were evident for seven of the nine markers measured (IL-8, IL-10, IL-13, IL-15, IL-18, iNOS, and LITAF). In all cases, HAS with probiotics exhibited significantly greater expression for all the inflammatory markers than the controls, while there was no difference between them in LAS.

For the thymus, significant line-by-treatment interactions involved IL-2, IL-8, and IL-10. LAS without probiotics had higher expression for IL-2, IL-8, and IL-10 than their probiotic counterparts. Both lines of chickens, when given probiotics, exhibited IL-2, IL-8, and IL-10 expressions that were intermediate to both lines without probiotics.

For the spleen, the three line-by-treatment interactions involved IL-13, IL-16, and iNOS. For IL-13, its expression was upregulated in both HAS and LAS with no probiotic supplementation. However, with probiotics, there was a greater IL-13 expression for LAS than HAS. In HAS, IL-16 was upregulated for chicks with no probiotics than the other groups. However, in LAS with probiotic IL-16, expression was significantly upregulated, as compared to LAS with no probiotics. For iNOS expression, there was a crossover interaction because LAS with probiotics and HAS without probiotics were both upregulated compared to their within-line counterparts.

## Discussion

The conundrum that involves interactions between a pathogen and its host is that variation in response to treatment can be complex and dynamic. Specifically, a pertinent question arises: why do some individuals get sick and may even die, while others show little, if any, outward response? Our results demonstrate this complexity. The chosen pathogen, *H. meleagridis*, is a very large extracellular unicellular protozoan eukaryote that exhibits a multitude of cytokine responses. Although generally lethal in turkeys, in chickens, symptoms range from mild to severe, including death (Beckstead, 2019). The two lines we chose for hosts originated from a common random bred founder population. Thus, the original gene pool had undergone long-term divergent selection for the single trait, antibody response to a nonpathogenic antibody, i.e., sheep red blood cells (Siegel and Gross, 1980; Lillie et al., 2017). These lines were known to respond differently to challenges of numerous pathogens (Gross et al., 1988; 2002; Sumners et al., 2012). The treatment involves the probiotic *L. reuteri*. Comparisons were based on cytokine responses in six tissues representing different primary germ layers. The results reflected multiple responses among tissues for cytokine responses and the complexity of treatment and genetic population for the tissue assayed.

The lack of cecal and hepatic lesions in our experiment might not be uncommon because histomoniasis is usually less severe in chickens than that in turkeys (McDougald, 2003). Egg-type chickens (ISA brown) may not show lesions typical of histomoniasis (Esquenet et al., 2003), and Powell et al. (2009) reported livers analyzed for *H. meleagridis* mRNA were all negative in broilers at 10 days post-challenge. Liebhart et al. (2011) and Chadwick et al. (2020) noted that histomoniasis in chickens was not as severe as in turkeys, based on the lower frequency of cecal lesions and mortality. Additionally, Chadwick et al. (2020) suspected that young breeder chickens may actually clear the *Histomonas* infection by 10 days post-challenge, as indicated by low to no cecal lesions, and very few if any hepatic lesions. Since *H. meleagridis* is an extracellular pathogen, it was not surprising that in six of the seven line comparisons, where there were no interactions, the expression was greater in LAS than that in HAS.

The brain is an organ where one would not expect to observe cytokine involvement associated with histomoniasis because of its primary origin. However, this assumption did not prove to be meaningful because responses of four cytokines, IL-2, IL-15, IL-18, and LITAF, were detected in the brain tissue. IL-18 and LITAF were upregulated in the brain of LAS chicks, and IL-2 and IL-15 were upregulated in the brain tissue of chicks when given the drinking water with the probiotic. In the HAS brain, LITAF was downregulated by the probiotic. Lillehoj et al. (2001) reported that both IL-15 and IL-2 function as T-cell growth factors and enhance type 1 cell-mediated immunity. The elevated IL-2 and IL-15 in the brain are possibly related to microglia that function as central nervous system macrophages derived from the monocyte/macrophage linage (Cuadros et al., 2006), which are involved in type 1 immune responses.

We confirmed the results reported by Lillehoj et al. (2001) that IL-15 was expressed in several tissues. However, they found that IL-2 was expressed only in concanavalin A-activated spleen cells *in vitro*. They also cite sources which indicate that IL-2 is expressed in activated T cells, which can be found in other tissues, and that IL-15 expression was enhanced in tissues where there was an invasion of mononuclear T cells, which are a characteristic of developing histomoniasis. Thus, our observation of extensive IL-2 expression in all tissues is further confirmation that *H. meleagrid*is had/was causing prolonged activation of T cells, as reported by Mitra et al. (2017), Kidane et al. (2018), and Lagler et al. (2019).

Highly reactive free radicals further damage the integrity of the liver cells, contributing to the enterohepatitis characteristic of histomoniasis. Another factor involved in mediation of host first-line defenses is the role of nitric oxide synthase (iNOS), which aids in the destruction of invading pathogens (Andrés et al., 2022). From iNOS, nitric oxide (NO) and potentially peroxynitrite anion (ONOO−) are produced as a part of regulated immune responses. Readily diffusible NO and ONOO− can cause DNA damage via oxidative deamination (Sharifi-Rad et al., 2020; Pérez de la Lastra et al., 2022). Both NO and ONOO− have short biological half-lives, allowing these damaging ionic forms to subdue, or even kill, invading pathogens at the site of infection (Radi et al., 2001; Prolo et al., 2014). Therefore, line-by-tissue interactions in both the liver and spleen suggest a role for iNOS to immunological response to *H. meleagridis*; however, the inconsistency across lines and treatments suggests caution in generalizations. Line-by-tissue interactions, for the range of inflammatory markers, reflect the complexity of genetic differences of background host populations in response to infection and suggest caution be exercised in generalizing results observed from only one genetic stock.

When one attempts to account for the genetic immune differences between HAS and LAS, one cannot overlook the major histocompatibility B complex genotypes. Hussain and Qureshi (1997) examined the potential for four macrophage cell lines to produce NO from increased iNOS expression after lipopolysaccharide stimulation *in vitro*. They found that chickens with different MHCB genotypes, GB1 (B13B13), GB2 (B6B6), Cornell K strain (B15B15), and MQ-NCSU (an unknown MHCB genotype), and cells, without LPS stimulation, produced minimal amounts of nitrite, a product of NO metabolism. However, on stimulation with LPS, GB1 and GB2 cell lines produced slightly elevated levels of nitrite, but MQ-NCSU and K-strain macrophages produced nearly four-fold more nitrite than did the GB1 and GB2 cells. Thus, the elevated hepatic iNOS expression in HAS, which is B21 at the MHC (Martin et al., 1986), with probiotics compared to HAS without probiotics and LAS with and without probiotics illustrated that other factors could be influencing iNOS in HAS. If our original hypothesis was confirmed, HAS should have been less resistant than LAS to *H. meleagridis*. Therefore, the upregulation of iNOS expression in the probiotic-treated HAS liver should be considered a biological response that required HAS to expend greater biological resources to survive the parasitic challenge compared to LAS, which was inherently more resistant/tolerant to the parasite.

Our experiment consisted of a disease challenge given to two lines of chickens that originated from a common founder population, and these lines were divergently selected for antibody response to a common antigen. The results show that differential expressions of immunologically associated cytokines vary among tissues assayed and the prior history of the selected lines. The frequency of the cytokine marker by treatment and by line interactions varied among tissues. They were more reflective of tissues from endodermal and mesodermal origins rather than the ectodermal origin. Responses to inflammatory markers that may be observed in tissues of one line may not be observed in another, reflecting parallel and/or co-evolution, depending on the pathogens and hosts.

## Data Availability

The original contributions presented in the study are included in the article/Supplementary Materials; further inquiries can be directed to the corresponding author.
